# Incidence of Acute Kidney Injury and Associated Mortality among Individuals with Drug-Susceptible Tuberculosis in Uganda

**DOI:** 10.34067/KID.0000000000000551

**Published:** 2024-08-14

**Authors:** Grace Kansiime, Abinet M. Aklilu, Joseph Baruch Baluku, Farah Yasmin, Michael Kanyesigye, Conrad K. Muzoora, F. Perry Wilson, Francis Bajunirwe, Ursula Brewster, Robert Kalyesubula

**Affiliations:** 1 Department of Internal Medicine, Mbarara University of Science and Technology, Mbarara, Uganda; 2 Section of Nephrology, Yale School of Medicine, New Haven, Connecticut; 3 Clinical and Translational Research Accelerator, Yale University, New Haven, Connecticut; 4 Division of Pulmonology, Kiruddu National Referral Hospital, Kampala, Uganda; 5 Global Health Collaborative, Mbarara University of Science and Technology, Mbarara, Uganda; 6 Department of Community Health, Mbarara University of Science and Technology, Mbarara, Uganda; 7 Department of Physiology and Department of Medicine, Makerere University College of Health Sciences, Kampala, Uganda

**Keywords:** AKI, HIV nephropathy, kidney disease

## Abstract

**Key Points:**

- AKI is thought to be a rare complication in patients with tuberculosis (TB) infection and is mostly attributed to TB drugs.
- Our findings show AKI occurs more often than previously thought and approximately 33% of patients with drug-susceptible TB may have kidney dysfunction.
- According to our study findings, monitoring kidney function should be routine among patients diagnosed with TB even before treatment initiation.

**Background:**

Although tuberculosis (TB) is associated with significant mortality and morbidity, its impact on kidney function is not well understood and is often attributed to anti-TB drugs. We aimed to assess the incidence of AKI in the immediate post-TB diagnosis period in Uganda, a TB/HIV-endemic country in sub-Saharan Africa.

**Methods:**

We included patients enrolled in an observational cohort study of adults diagnosed with drug-susceptible TB followed longitudinally. Adults (≥18 years) without known kidney disease were enrolled between August 2022 and July 2023 at three regional hospitals serving 12.5% of the Ugandan population. Our primary outcome was incidence of Kidney Disease Improving Global Outcomes-defined AKI within 2 weeks of TB diagnosis. Other outcomes included predictors of AKI and its association with 30-day survival.

**Results:**

A total of 156 adults were included. The median age was 39 (interquartile range, 28–53) years, most patients were male (68.6%), and 49.4% had HIV. People with HIV had shorter time to TB diagnosis from symptom onset (21 days; interquartile range, 7–30) compared with HIV-negative participants (60 days; interquartile range, 23–90), *P* < 0.001. The incidence of AKI was 33.3% (52/156) and was similar between people with and without HIV. Proteinuria or hematuria at enrollment was associated with higher odds of AKI (odds ratio, 2.68; 95% confidence interval, 1.09 to 6.70; *P* approximately 0.033). AKI was associated with a significant risk of mortality (adjusted hazard ratio, 8.22; 95% confidence interval, 1.94 to 34.72; *P* approximately 0.004) independent of HIV status.

**Conclusions:**

The overall incidence of AKI in the immediate post-TB diagnosis period is high and associated with increased mortality risk. Our findings suggest that monitoring kidney function should be routine for patients with TB, including before treatment initiation.

## Visual Abstract

**Figure:**
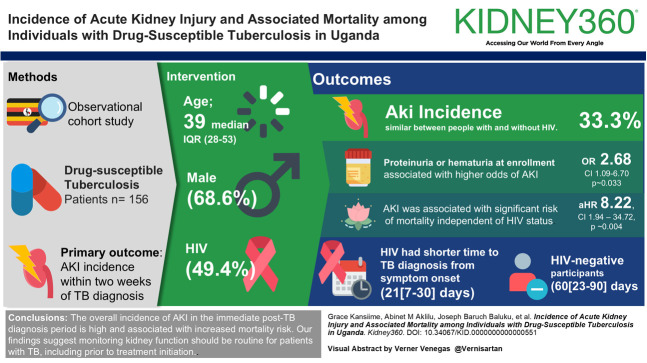

## Introduction

AKI, an abrupt decline in kidney function occurring within 7 days, is common and associated with poor outcomes.^1^ It is estimated that 85% of people with AKI live in low- and middle-income countries (LMICs) where diagnostic and therapeutic options are limited.^2^ Contrary to the experience in high-income countries, infectious diseases are major contributors to the development of AKI and CKD in LMICs.^3–6^ Tuberculosis (TB) is particularly a major public health concern and contributes to significant morbidity and mortality.^7,8^ Incident AKI causes challenges with drug dosing, affecting patient management by limiting treatment options for the primary disease.

AKI is associated with an estimated two million deaths per year globally,^9^ with higher incidence of poor outcomes in LMICs compared with high-income countries.^10^ For severe AKI requiring dialysis, patient mortality exceeds 90% because of the limited access to such life-saving resources in LMICs.^11–13^ AKI survivors are at an increased risk of developing CKD and ESKD—both conditions carrying a high societal, personal, and economic burden.^1,14,15^ Up to 13% of individuals who experience AKI in sub-Saharan Africa (SSA) develop CKD.^16^

There were approximately 10.6 million TB cases in 2021 globally,^17^ and SSA contributes up to a quarter of these cases.^18^ Uganda, in particular, has been reclassified by the World Health Organization as both a TB and TB/HIV high-burdened country with approximately 90,000 cases in 2020, 30,000 coinfected with HIV, and 7400 deaths.^19^ Although AKI was thought a rare complication of TB,^20–22^ it has been recently shown to occur commonly at an incidence of about 10.3%.^23^ In addition to TB drugs,^15,20,24^ HIV coinfection may increase the likelihood of AKI among patients with TB.^25,26^

Approximately 1/3 of patients with TB in SSA have HIV coinfection,^27^ and more than half of AKI events among HIV-infected patients are associated with opportunistic infections.^28,29^ Volume depletion, sepsis, liver disease, comorbidities, and antiretroviral toxicity further increase the risk of developing AKI in patients with TB/HIV coinfection.^30,31^

Hence, TB/HIV coinfection can be expected to synergistically increase AKI incidence. There is a lack of data comparing the spectrum and trajectory of kidney function among patients with TB in Uganda, where kidney function monitoring is not routine, because of limited resources. We aimed to prospectively assess the incidence of AKI in the immediate period of TB diagnosis and treatment initiation, its predictors, and its association with 30-day mortality among patients with drug-susceptible TB (DS-TB) with or without HIV coinfection in Uganda, a country with high burden of TB/HIV in SSA.

## Methods

### Study Setting and Participants

Data for this analysis come from the Spectrum of Kidney Disease among Patients with TB study, an ongoing observational cohort study of adults with DS-TB with and without HIV coinfection. The study is carried out at three Regional Referral Hospitals (RRHs) in rural Uganda (Mbarara, Masaka, and Kabale). The Masaka and Kabale RRHs each serve an estimated population of two million while the Mbarara RRH serves four million people in urban, periurban, and most rural catchment areas.

### Eligibility Criteria

Adults (≥18 years) recently diagnosed with TB (with or without HIV coinfection) at one of the three sites were included. Exclusion criteria included having a history of CKD, being on TB treatment for more than 7 days, being diagnosed with multidrug resistant TB as described below, being unable to follow-up at one of the three sites, and inability to provide informed consent.

### Definitions

AKI was defined according to the Kidney Disease Improving Global Outcomes serum creatinine (sCr) criteria as a 50% relative increase in sCr from an imputed baseline at day 0 or a 50% relative increase in sCr at day 7 compared with day 0, with those not diagnosed with AKI on day 0. Baseline creatinine imputation was performed so as not to discount kidney dysfunction at the time of enrollment in patients with no known history of CKD because none of the participants had a sCr measured before enrollment and given the age and comorbidity profile of the participants. Baseline creatinine was imputed from an eGFR of 75 ml/min based on the Acute Dialysis Quality Initiative recommendations^1^ using the age and sex of the participant in the CKD Epidemiology Collaboration eGFR equation.^32^ AKI stage was defined according to the Kidney Disease Improving Global Outcomes criteria where stage 1 is an increase of 1.5 to <2 times the baseline creatinine, stage 2 an increase of 2 to <3 times the baseline creatinine, and stage 3 an increase by ≥3 times the baseline creatinine or need for dialysis. We did not consider the urine output criteria.

DS-TB was defined as bacteriologically confirmed or clinically diagnosed case of TB without evidence of infection with strains resistant to rifampicin and isoniazid (INH).^33^ This is done routinely at diagnosis, and once the GenXpert is positive, sensitivity testing to rifampin (RIF) and INH drugs is done before treatment initiation.

### Study Procedure

All participants diagnosed with TB during the study period at the three RRHs were screened for enrollment within 7 days of TB treatment initiation. According to Ugandan guidelines,^34^ TB is diagnosed bacteriologically using smear microscopy; culture, World Health Organization-recommended molecular TB diagnostics (Xpert MTB/RIF), or clinically by a medical worker based on suggestive clinical symptoms; and chest x-ray abnormalities.

Detailed history, including baseline demographics, comorbidities, medication, social history, duration of symptoms, TB and HIV-related treatment, and potential nephrotoxic exposure history, were collected at the time of enrollment. We collected urine samples for urinalysis and a blood sample for a complete blood count (Sysmex XN550), sCr, and urea (Humastar 200). Data about HIV status, clusters of differentiation 4 (CD4), and viral load were abstracted from the participants' medical records. Enrolled participants had a follow-up clinic visit on day 7 after enrollment where they completed a questionnaire (Supplemental Figure 1) to assess for risk factors of AKI and underwent anthropometric measurements (height and weight), creatinine measurement, and urinalysis. Participants had sCr measured at enrollment and 7 days after enrollment, if alive. The participant or their next of kin were given a follow-up phone call on day 30 to establish their vital status.

We used Research Electronic Data Capture (Vanderbilt University), a cloud-based secure, HIPAA-compliant system, for data collection and management.^35^

All patients were prescribed a standard anti-TB regimen of daily INH, RIF, ethambutol, and pyrazinamide for the first 2 months, followed by daily INH and RIF for 4 months according to the Uganda TB treatment guidelines.^34^ All medications were fixed drug combinations, and treatment was offered under programmatic conditions by the hospital staff. As such, our study team did not interfere with patients' treatment. However, copies of all investigation results (complete blood count, kidney function tests, urinalysis, and ultrasound scan) performed by the study were made available to the primary care teams to assist in patient management.

### Covariates

Variables in the descriptive analyses included baseline demographics (age, sex), distance from the hospital, hospital admission status, comorbidities (hypertension, diabetes mellitus, HIV infection), duration of TB symptoms at the time of enrollment, pulmonary versus extrapulmonary TB, body mass index, sCr level at enrollment, education level, residence area type, and components of the complete blood count, BUN, and urinalysis findings, such as specific gravity, proteinuria, and hematuria.

### Outcomes

The primary outcome of this study was the cumulative incidence of AKI assessed at enrollment (baseline—day 0) and 7 days postenrollment (day 7). Secondary outcomes included a comparison of AKI incidence between people with and without HIV, predictors of AKI, and the association of AKI with 30-day survival stratified by TB/HIV coinfection status.

### Statistical Analysis

Descriptive data were summarized as median and interquartile range (IQR) for continuous variables if skewed and mean and SD if normally distributed. Categorical variables were presented as counts and percentages. Baseline characteristics were further compared between participants with TB/HIV coinfection and those without HIV using the Wilcoxon rank-sum test for continuous variables and the chi-squared test for categorical variables.

The incidence of AKI was computed as the total percentage of participants with a 50% relative increase in sCr from baseline at day 0 or a 50% relative increase in sCr at day 7 compared with day 0.

We performed logistic regression to identify predictors of AKI and mortality. To identify predictors of AKI, we included only the patients who had no AKI at the time of enrollment and had creatinine measured at day 7. We selected variables hypothesized as most relevant predictors on the basis of domain knowledge. Correlated variables were combined into single measure to avoid collinearity. For multivariable logistic regression analyses, we used the rule of ten events per one predictor variable to avoid overfitting.

Mortality was defined as death from any cause during the 30-day follow-up period. In the mortality analysis where AKI is the exposure to avoid immortal time bias as we could not perform a time-varying exposure analysis, we performed landmark analysis of mortality up to 30 days from AKI where we only included those who survived to 7 days from enrollment. All of the participants included in this analysis were alive at day 7, had a sCr at day 7, and did not meet AKI criteria at enrollment. The presence of AKI at day 7 (the start of follow-up for this analysis) defined the exposure group. Risk of mortality was assessed using multivariable Cox proportional hazards regression analysis. Survival curves were generated using the Kaplan–Meier method.

Statistical significance was defined as a two-tailed *P* value of <0.05. Statistical analysis was performed using R Studio version 4.1.0. We used the Strengthening the Reporting of Observational Studies in Epidemiology cohort checklist when writing our report.^36^

### Ethical Considerations

The protocol received ethical approval from Mbarara University of Science and Technology research and ethics committee (MUST-2022-389) and Uganda National Council for Science and Technology (HS2357ES). All study participants gave written informed consent to participate in the study.

## Results

**Figure 1:**
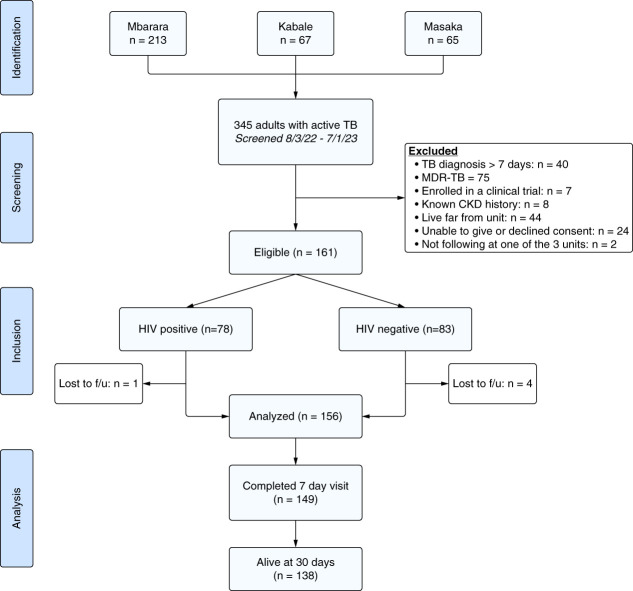
**Study flow diagram.** f/u, follow up; MDR TB, multi-drug resistant TB; TB, Tuberculosis.

Between August 2022 and July 2023, we screened 345 patients with TB for eligibility. A total of 161 participants (78 with TB/HIV coinfection and 83 non-HIV) were eligible for enrollment. Five participants were excluded for incomplete data leaving 156 participants whose data were analyzed. The Strengthening the Reporting of Observational Studies in Epidemiology study flow diagram is shown in Figure 1.

### Baseline Characteristics

Baseline demographics are presented in Table 1. The median time from TB diagnosis to enrollment was 1 (IQR, 0–1) day and no different by HIV status. Overall, the median age of the cohort was 39 (IQR, 28–53) years, 68.6% were male, and 49.4% had HIV coinfection. Of those who had HIV, 31 (39.7%) were newly diagnosed at the time of TB diagnosis. The rest were already known to be people with HIV. The median body mass index was 18.2 (IQR, 16.4–20.2) kg/m^2^, and 82 of 156 (52.6%) were underweight.

**Table 1:** Baseline characteristics of the study participants

Characteristic	Total, *n*=156	HIV+, *n*=77	HIV−, *n*=79	*P* Value
**Baseline demographics**				
Age, median (IQR)	39 (28–53)	40 (32–52)	32 (27–55)	0.137
Sex, *No.* (%)				0.007
*Female*	49 (31.4)	32 (41.6)	17 (21.5)	
*Male*	107 (68.6)	45 (58.4)	62 (78.5)	
≤Primary education, *No.* (%)	91 (58.3)	51 (65.4)	40 (53.0)	0.048
Distance from hospital, km, median (IQR)	20.0 (6.0–41.5)	15.0 (6.0–40.0)	20.0 (6.0–42.0)	0.978
Residence type, *No.* (%)				0.592
*Rural*	78 (50.0)	41 (51.9)	37 (48.1)	
*Suburban*	20 (12.8)	8 (10.1)	12 (15.6)	
*Urban*	58 (37.2)	30 (38.0)	28 (36.4)	
**Baseline comorbidities**				
HTN, *No.* (%)	4 (2.6)	0	4 (5.1)	0.120
DM, *No.* (%)	7 (4.5)	1 (1.3)	6 (7.6)	0.117
HIV specific				
*On HAART, No. (%)*		34 (43.0)		
TDF containing, *No.* (%)		26 (32.9)		
*CD4 count, median (IQR)*		87.0 (43.0–218.0)		
CD4 <200, *No.* (%)		*54 (68.4)*		
BMI, median (IQR), kg/m^2^	18.2 (16.4–20.2)	18.8 (16.9–20.2)	17.7 (15.9–20.1)	0.136
BMI categories, kg/m^2^, *No.* (%)				0.116
*<18.5*	82 (52.6)	35 (45.5)	47 (59.5)	
*18.5–25*	65 (41.7)	38 (49.3)	27 (34.2)	
*>25*	9 (5.8)	4 (5.2)	5 (6.3)	
**TB symptoms, *No.* (%)**				
Cough	137 (88.2)	61 (79.2)	76 (96.2)	0.001
Hemoptysis	20 (14.1)	5 (6.5)	15 (19.0)	0.057
Dyspnea	62 (39.8)	26 (33.8)	36 (45.6)	0.132
Weight loss	142 (91.3)	69 (89.6)	73 (92.4)	0.541
Symptom duration, d, median (IQR)	30.0 (14.0–64.0)	21.0 (7.0–30.0)	60.0 (23.0–90.0)	<0.001
**TB category, *No.* (%)**				0.729
Pulmonary	144 (92.3)	70 (90.9)	74 (93.7)	
Extrapulmonary^a^	12 (7.7)	7 (9.0)	5 (6.3)	
**Weight loss in the past month, kg, *No.* (%)**				0.464
≤2	25 (16.0)	15 (19.5)	10 (12.7)	
2–5	44 (28.2)	22 (28.6)	22 (27.8)	
>5	87 (55.8)	40 (51.9)	47 (59.5)	
Time since TB diagnosis, d, median (IQR)	1 (0–1)	1 (0–1)	1 (0–2)	0.941
Time since TB Tx start, d, median (IQR)	0 (0–1)	0 (0–1)	0 (0–1)	0.719
**Vitals, mm Hg, median (IQR)**				
SBP	106 (97–120)	103 (96–119)	109 (99–121)	0.122
DBP	70 (63–77)	69 (62–77)	71 (65–78)	0.128
HR	101 (90–118)	101 (92–118)	101 (88–117)	0.405
SpO_2_, %, median (IQR)	97 (95–98)	98 (96–98)	97 (94–98)	0.212
**Laboratory values, median (IQR)**				
Hgb, g/dl	11.3 (9.2–14.0)	10.7 (8.7–13.8)	11.5 (9.3–14.1)	0.458
WBC×1000/*μ*l	6.2 (4.3–8.9)	4.9 (3.7–7.7)	7.7 (5.5–10.7)	<0.001
*Neutrophils, %*	62.7 (48.2–74.3)	58.1 (43.3–73.1)	67.2 (51.4–75.6)	0.046
*Lymph, %*	24.4 (15.2–36.6)	26.8 (16.7–40.3)	21.3 (14.5–34.5)	0.076
*Eos, %*	1.2 (0.3–2.7)	1.2 (0.3–2.7)	1.1 (0.3–2.6)	0.790
*Mono, %*	8.9 (6.1–11.9)	8.8 (5.8–11.6)	9.2 (6.2–12.4)	0.324
PLT×1000/*μ*l	249.5 (178.8–389.5)	227.0 (172.0–329.0)	299.0 (197.0–436.5)	0.008
**Kidney function at enrollment**				
sCr, mg/dl, median (IQR)	0.86 (0.70–1.09)	0.90 (0.76–1.20)	0.80 (0.64–0.99)	0.009
eGFR, ml/min, median (IQR)	104.3 (77.9–122.9)	89.9 (69.9–111.8)	110.5 (94.2–129.5)	<0.001
Hospitalized at enrollment, *No.* (%)	76 (48.7)	47 (61.0)	29 (36.7)	0.002
**Enrollment sites, *No.*** **(%)**				0.390
Mbarara	116 (74.5)	61 (79.5)	55 (69.9)	
Kabale	20 (12.4)	8 (10.3)	12 (14.5)	
Masaka	20 (13.0)	8 (10.3)	13 (15.7)	

BMI, body mass index; CD4, clusters of differentiation 4; DBP, diastolic BP; DM, diabetes mellitus; HAART, highly active antiretroviral therapy; Hgb, hemoglobin; HR, heart rate; HTN, hypertension; IQR, interquartile range; PLT, platelet count; sCr, serum creatinine; SBP, systolic BP; SpO_2_, saturation of peripheral oxygen; TB, tuberculosis; TDF, tenofovir disoproxil fumarate; Tx, treatment; WBC, white blood cell count.

a Tuberculosis adenitis and abdominal tuberculosis.

Compared with the non-HIV group, participants in the TB/HIV coinfection group were older (median age 40 versus 32 years) and more likely to be female (41.6% versus 21.5%). Participants with TB/HIV coinfection had a median CD4 count of 87.0 (IQR, 43.0–218.0) cells/mm^3^, 54 of 77 (68.4%) had a CD4 count below 200 cells/mm^3^, and 43% were on antiretroviral therapy. Those with TB/HIV coinfection had a lower eGFR at enrollment with a median eGFR of 89.9 (IQR, 69.9–111.8) ml/min compared with 110.5 (IQR 94.2–129.5) ml/min among those without HIV. Participants in the non-HIV group were more likely to have had symptoms for a longer duration before diagnosis with median of 60 (IQR, 23–90) days compared with 21 (IQR, 7–30) days in those with TB/HIV coinfection. The rest of the baseline characteristics were similar between the two groups.

### Incidence of AKI

The incidence of AKI within 1 week of study enrollment was 33.3% (52/156). Twenty-seven individuals (16.7%) had AKI at the time of enrollment. Of those who completed 7-day follow-up (*n*=149), an additional 25 had developed AKI at follow-up. The median creatinine at the time of AKI was 1.51 (IQR, 1.23–1.93) mg/dl. Most patients who died (17/18) within 30 days of enrollment were hospitalized.

### Secondary Outcomes

Secondary outcomes by TB/HIV coinfection status are shown in Table 2. There was no difference in the incidence of AKI between participants with or without HIV coinfection (33.8 versus 32.9%). Of those with AKI, 37 of 52 (71.2%) had stage 1 AKI, 12 (23.1%) had stage 2 AKI, and 3 (5.8%) had stage 3 AKI at the time of diagnosis. Of those with AKI at enrollment, 18 of 27 (66.7%) had some improvement at day 7 of follow-up, 2 (7.4%) had AKI progression to a higher stage at day 7, 2 (7.4%) had died before reaching the day 7, and 5 (18.5%) had no change in their creatinine by day 7 follow-up (Supplemental Figure 2).

**Table 2:** Incidence of AKI and 30-day mortality in tuberculosis and tuberculosis/HIV coinfected individuals

Variable	Total, *n*=156	HIV+, *n*=77	HIV−, *n*=79	*P* Value
AKI, *No.* (%)	52 (33.3)	26 (33.8)	26 (32.9)	0.910
Stage 1, *No.* (%)	37 (71.2)	18 (69.2)	19 (73.1)	0.835
Stage 2, *No.* (%)	12 (23.1)	6 (23.1)	6 (23.1)	
Stage 3, *No.* (%)	3 (5.8)	2 (7.7)	1 (3.8)	
sCr at AKI, median (IQR), mg/dl^a^	1.51 (1.23–1.93)	1.46 (1.21–1.80)	1.55 (1.26–1.99)	0.528
Died in 7 d, *No.* (%)	7 (4.5)	2 (2.6)	5 (6.3)	0.460
Died in 30 d, *No.* (%)	18 (11.5)	7 (9.1)	11 (13.9)	0.488

IQR, interquartile range; sCr, serum creatinine.

a Among patients with AKI.

Overall, 15 participants had indications for dialysis (renal failure refractory to conservative medical management), but none of them were able to undergo dialysis because of cost and access issues. They were managed conservatively, and 8 (53.3%) died.

Characteristics of patients with and without AKI are shown in Supplemental Table 1. Those who developed AKI were more likely to have proteinuria and hematuria on urinalysis and granular casts on urine microscopy.

All participants, 77 in the TB/HIV coinfection group, and 79 in the non-HIV group completed 30-day vital status follow-up or were known to have died during this period. Seven (4.5%) of all participants (*n*=156) had died within 7 days of enrollment, and a total of 18 of 156 (11.5%) had died within 30 days of enrollment. There was no difference in mortality incidence between the two groups. Most of the patients who died (17/18) within 30 days of enrollment were hospitalized.

#### Predictors of AKI

Among those without AKI at enrollment, adjusted for age and HIV, having any degree of proteinuria or hematuria was associated with higher odds of AKI at day 7 (odds ratio [OR], 2.68; 95% confidence interval [CI], 1.09 to 6.70; *P* approximately 0.033). There was no statistically significant association between AKI at 7 days and other baseline covariates, including HIV, TB treatment duration, symptom duration, admission status, baseline demographics, and vitals.

#### Survival Analyses

**Figure 2:**
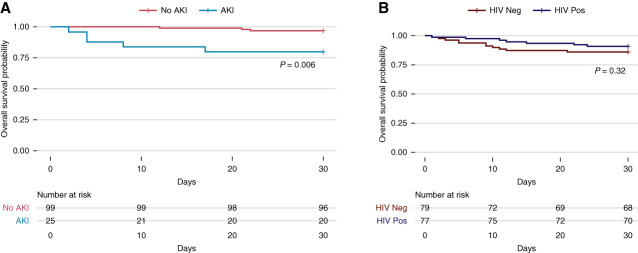
**Survival curves.** (A) AKI as a predictor for mortality; (B) HIV as a predictor for mortality.

A total of 124 participants who survived to 7 days, had a follow-up creatinine at day 7, and did not have AKI on day 0 were included in the survival analysis with AKI as the exposure. Participants who developed AKI had a significantly higher risk of 30-day mortality compared with those without AKI on unadjusted analysis (hazard ratio [HR], 7.49; 95% CI, 1.79 to 31.37; *P* approximately 0.006) and adjusted for HIV status (HR, 8.22; 95% CI, 1.94 to 34.72; *P* approximately 0.004) (Figure 2A and Table 3).

**Table 3:** Risk of mortality in patients with tuberculosis within 30 days of follow-up

Exposures	HR (95% CI)	*P* Value
**HIV**		
Unadjusted	0.62 (0.25 to 1.60)	0.324
Adjusted^a^	0.35 (0.14 to 0.91)	0.031
**AKI**		
Unadjusted	7.49 (1.79 to 31.37	approximately 0.006
Adjusted^b^	8.22 (1.94 to 34.72)	approximately 0.004

CI, confidence interval; HR, hazard ratio; TB, tuberculosis.

a Adjusted for hospitalization status.

b Adjusted for HIV status.

HIV coinfection at enrollment was associated with lower mortality. Although the association was not statistically significant on unadjusted analysis (HR, 0.62; 95% CI, 0.25 to 1.60; *P* = 0.324), it became significant on adjusting for inpatient status (adjusted HR, 0.35; 95% CI, 0.14 to 0.91; *P* approximately 0.031) (Table 3). Unadjusted Kaplan–Meier survival curves are shown in Figure 2.

#### Predictors of Mortality

Adjusted for HIV status and age, being hospitalized at enrollment (OR, 33.93; 95% CI, 6.37 to 630.4; *P* < 0.001) and AKI (OR, 8.04; 95% CI, 2.32 to 37.22; *P* = 0.002) were independently associated with significantly higher odds of mortality within 30 days. Adjusted for HIV status, higher oxygen saturation (saturation of peripheral Oxygen) at enrollment was associated with lower odds of mortality with an OR, 0.84; 95% CI, 0.74 to 0.92; *P* = 0.002. Adjusted for inpatient status at enrollment, HIV coinfection was associated with lower odds of mortality (OR, 0.32; 95% CI, 0.10 to 0.92; *P* = 0.039).

## Discussion

In this prospective observational cohort study of patients recently diagnosed with DS-TB conducted at three RRHs in rural Uganda, we found a high incidence of AKI in the immediate period of TB diagnosis. Our results show a three times higher incidence of AKI compared with that shown in a prospective study of a Taiwanese cohort.^23^ The lack of kidney function assessment in the first week and exclusion of patients with evidence of volume depletion, hypotension, and nephrotoxin exposure could have contributed to the lower AKI incidence in that study. Other studies have been retrospective and/or in specific populations, such as those with TB/HIV coinfection that were limited by the lack of protocolized creatinine measurement in low-resource settings.^15,37,38^ AKI in TB has mainly been attributed to TB drugs.^20–24,39^ Our study evaluated AKI within the first 1–2 weeks of diagnosis and treatment initiation. The high incidence of AKI as early as on enrollment (*i.e*., a median of 1 day from diagnosis) in this TB treatment-naïve cohort contradicts previous hypotheses attributing AKI in patients with TB mostly to TB medications.

There was no difference in AKI incidence between participants with and without HIV. Furthermore, on adjusted analysis, those with HIV had lower mortality than those with TB alone. This is surprising, especially as most patients with HIV had poorly controlled HIV with low CD4 counts. Previous studies have shown a two- to three-fold greater incidence of AKI among people living with HIV compared with those without HIV.^3,6,40^ Those studies, however, have been among patients who were acutely ill. The current Ugandan HIV care policy is test and treat where patients are started on antiretroviral therapy at HIV diagnosis irrespective of level of immunosuppression.^41^ People living with HIV are actively screened for TB routinely at diagnosis or first symptom/suspicion during care using tests, such as urine lipoarabinomannan, which facilitates early TB diagnosis in these patients compared with people without HIV. This is supported by our finding that HIV-positive participants had shorter symptom duration at the time of diagnosis than HIV-negative participants. Participants without HIV were diagnosed late and were sicker in this cohort. Previous studies done especially in our setting have not had comparative groups. These factors may partially explain why the incidence of AKI is comparable between those with and without HIV coinfection and suggest that public health services directed at HIV-positive individuals might have broad benefits.

We found that having proteinuria or hematuria was a significant predictor of AKI in this study. Proteinuria and hematuria have been reported to be prevalent and significantly associated with AKI and have been used in developing a predictive model for AKI among patients with TB.^39,42,43^ Moreover, these signs of kidney injury may appear before creatinine elevation and have been suggested as earlier markers of AKI.^44^ This warrants further investigation particularly in resource-limited settings where urinalysis is more easily accessible and cost-effective to implement on a broad scale.

In this Ugandan cohort of patients with recent diagnosis of TB, we show that AKI in this setting is associated with a more than seven-fold higher risk of mortality. This is not surprising because AKI has been consistently shown to be an independent risk factor of mortality in multiple cohorts of patients with various degrees of illness.^45–49^

Our study has some limitations which should be taken into consideration. We were unable to ascertain the cause of AKI at the time of diagnosis (as we could not rule out confounders like herbal medication or other nephrotoxin use and other organ failures) and monitor kidney function daily in the first 7 days of follow-up. In addition, a major limitation is the lack of baseline kidney function, which might have led to underestimation of the incidence of AKI in this cohort with no history of CKD. This was despite the majority of the patients having had follow-up in HIV clinic or prior hospitalization. To mitigate this challenge, we used the conservative imputation technique using an eGFR of 75 ml/min. We further assumed normal baseline kidney function if the patient did not disclose a history of CKD. Moreover, our primary outcome, AKI, was defined and studied using only sCr criteria because we were unable to use the urine output criteria or early biomarkers of AKI. Nonetheless, we used surrogate baselines for all patients because we did not have sCr values before the TB diagnosis. This further underscores the importance of kidney function screening and monitoring in LMICs. Furthermore, we were unable to specifically diagnose/confirm renal/urological TB, and this may have confounded the urinary findings. The sample size as well was limited to fully assess predictors of AKI and mortality.

Despite these limitations, to the best of our knowledge, our study is the largest longitudinal prospective cohort study examining AKI incidence and short-term mortality among treatment-naïve patients with TB in a resource-limited setting. Although primary care guidelines exist for management of TB, AKI screening has not been emphasized and kidney function tests or urinalysis are not routinely performed at TB diagnosis or initiation of treatment^34^ because of lack of funding and awareness. Our data reinforce the importance of improving regular kidney function monitoring for patients diagnosed with TB, targeting early identification and management. This is particularly important for patients hospitalized at diagnosis of TB irrespective of HIV status. The inclusion of both hospitalized and nonhospitalized patients and those with and without HIV coinfection makes it generalizable to the population of patients with DS-TB in the region.

In summary, we found a significantly higher incidence of AKI among adults with DS-TB within the immediate period of diagnosis and treatment initiation in Uganda. We found that AKI is a strong risk factor of short-term mortality. These findings were stable after adjusting for HIV status. Future work in this area should focus on the longitudinal kidney function, underlying mechanisms of AKI and early mortality predictors to improve these outcomes in this patient population. Further larger prospective studies are urgently needed to understand the causes of AKI in this cohort and the implications of early kidney dysfunction on TB management. The high observed mortality indicates the importance of kidney function monitoring and urgency for immediate care and referral of patients with kidney dysfunction at the time of TB diagnosis.

## Supplementary Material

SUPPLEMENTARY MATERIAL

## Data Availability

All datasets used in this analysis may be shared through direct contact with the corresponding author on reasonable request. By recommendation of the Uganda National Council of Science and Technology, it is required to explain the aim of the requested information. The information will be shared respecting the confidentiality of the patients included in the study.

## Supplemental Material

Supplemental Table 1. Characteristics of participants with AKI.

Supplemental Figure 1. Follow-up data collection form.

Supplemental Figure 2. Trajectory of participants' serum creatinine from baseline to follow-up day 7.
