# Supplementary material for: Incidence of Acute Kidney Injury and Associated Mortality among Individuals with Drug-Susceptible Tuberculosis in Uganda
Source: Kidney360. 2024 Aug 14;5(10):1446–54. doi: 10.34067/KID.0000000000000551 (PMC11556924; doi:10.34067/KID.0000000000000551)
Supplement: SUPPLEMENTARY MATERIAL [file kidney360-5-1446-s001.pdf]

ASN
American Society of Nephrology
JASN
Journal of the American Society of Nephrology
KIDNEY360
Accessing Our World From Every Angle
CJASN
Clinical Journal of the American Society of Nephrology

ASN Journal Disclosure Form

As per ASN journal policy, I have disclosed any financial relationships or commitments I have held in the past 36 months as included below. I have listed my Current Employer below to indicate there is a relationship requiring disclosure. If no relationship exists, my Current Employer is not listed.

A. Aklilu reports the following:
Employer: Yale University School of Medicine

I understand that the information above will be published within the journal article, if accepted, and that failure to comply and/or to accurately and completely report the potential financial conflicts of interest could lead to the following: 1) Prior to publication, article rejection, or 2) Post-publication, sanctions ranging from, but not limited to, issuing a correction, reporting the inaccurate information to the authors' institution, banning authors from submitting work to ASN journals for varying lengths of time, and/or retraction of the published work.

Name: Abinet Mathias Aklilu
Manuscript ID: K360-2024-000147R1
Manuscript Title: Incidence of Acute Kidney Injury and Associated Mortality among Individuals with Drug-Susceptible Tuberculosis in Uganda
Date of Completion: May 24, 2024
Disclosure Updated Date: May 24, 2024

ASN
American Society of Nephrology
JASN
Journal of the American Society of Nephrology
KIDNEY360
Accessing Our World From Every Angle
CJASN
Clinical Journal of the American Society of Nephrology

ASN Journal Disclosure Form

As per ASN journal policy, I have disclosed any financial relationships or commitments I have held in the past 36 months as included below. I have listed my Current Employer below to indicate there is a relationship requiring disclosure. If no relationship exists, my Current Employer is not listed.

F. Bajunirwe has nothing to disclose.

I understand that the information above will be published within the journal article, if accepted, and that failure to comply and/or to accurately and completely report the potential financial conflicts of interest could lead to the following: 1) Prior to publication, article rejection, or 2) Post-publication, sanctions ranging from, but not limited to, issuing a correction, reporting the inaccurate information to the authors' institution, banning authors from submitting work to ASN journals for varying lengths of time, and/or retraction of the published work.

Name: Francis Bajunirwe
Manuscript ID: K360-2024-000147R2
Manuscript Title: Incidence of Acute Kidney Injury and Associated Mortality among Individuals with Drug-Susceptible Tuberculosis in Uganda
Date of Completion: August 9, 2024
Disclosure Updated Date: August 9, 2024

ASN
American Society of Nephrology
JASN Journal of the American Society of Nephrology
KIDNEY360 Accessing Our World From Every Angle
CJASN Clinical Journal of the American Society of Nephrology

ASN Journal Disclosure Form

As per ASN journal policy, I have disclosed any financial relationships or commitments I have held in the past 36 months as included below. I have listed my Current Employer below to indicate there is a relationship requiring disclosure. If no relationship exists, my Current Employer is not listed.

J. Baluku reports the following:
Employer: Kiruddu hospital; and Ownership Interest: Pfizer, JnJ, Nvidia, Apple.

I understand that the information above will be published within the journal article, if accepted, and that failure to comply and/or to accurately and completely report the potential financial conflicts of interest could lead to the following: 1) Prior to publication, article rejection, or 2) Post-publication, sanctions ranging from, but not limited to, issuing a correction, reporting the inaccurate information to the authors' institution, banning authors from submitting work to ASN journals for varying lengths of time, and/or retraction of the published work.

Name: Joseph Baluku
Manuscript ID: K360-2024-000147R1
Manuscript Title: Incidence of Acute Kidney Injury and Associated Mortality among Individuals with Drug-Susceptible Tuberculosis in Uganda
Date of Completion: May 24, 2024
Disclosure Updated Date: May 24, 2024

ASN American Society of Nephrology | JASN Journal of the American Society of Nephrology | KIDNEY360 Accessing Our World From Every Angle | CJASN Clinical Journal of the American Society of Nephrology

ASN Journal Disclosure Form

As per ASN journal policy, I have disclosed any financial relationships or commitments I have held in the past 36 months as included below. I have listed my Current Employer below to indicate there is a relationship requiring disclosure. If no relationship exists, my Current Employer is not listed.

U. Brewster reports the following:
Employer: Yale University School of Medicine

I understand that the information above will be published within the journal article, if accepted, and that failure to comply and/or to accurately and completely report the potential financial conflicts of interest could lead to the following: 1) Prior to publication, article rejection, or 2) Post-publication, sanctions ranging from, but not limited to, issuing a correction, reporting the inaccurate information to the authors' institution, banning authors from submitting work to ASN journals for varying lengths of time, and/or retraction of the published work.

Name: Ursula C. Brewster
Manuscript ID: K360-2024-000147R1
Manuscript Title: Incidence of Acute Kidney Injury and Associated Mortality among Individuals with Drug-Susceptible Tuberculosis in Uganda
Date of Completion: May 24, 2024
Disclosure Updated Date: February 8, 2024

ASN
American Society of Nephrology
JASN
Journal of the American Society of Nephrology
KIDNEY360
Accessing Our World From Every Angle
CJASN
Clinical Journal of the American Society of Nephrology

ASN Journal Disclosure Form

As per ASN journal policy, I have disclosed any financial relationships or commitments I have held in the past 36 months as included below. I have listed my Current Employer below to indicate there is a relationship requiring disclosure. If no relationship exists, my Current Employer is not listed.

R. Kalyesubula reports the following:
Employer: Makerere University

I understand that the information above will be published within the journal article, if accepted, and that failure to comply and/or to accurately and completely report the potential financial conflicts of interest could lead to the following: 1) Prior to publication, article rejection, or 2) Post-publication, sanctions ranging from, but not limited to, issuing a correction, reporting the inaccurate information to the authors' institution, banning authors from submitting work to ASN journals for varying lengths of time, and/or retraction of the published work.

Name: Robert Kalyesubula
Manuscript ID: K360-2024-000147R1
Manuscript Title: Incidence of Acute Kidney Injury and Associated Mortality among Individuals with Drug-Susceptible Tuberculosis in Uganda
Date of Completion: May 24, 2024
Disclosure Updated Date: May 24, 2024

ASN American Society of Nephrology | JASN Journal of the American Society of Nephrology | KIDNEY360 Accessing Our World From Every Angle | CJASN Clinical Journal of the American Society of Nephrology

ASN Journal Disclosure Form

As per ASN journal policy, I have disclosed any financial relationships or commitments I have held in the past 36 months as included below. I have listed my Current Employer below to indicate there is a relationship requiring disclosure. If no relationship exists, my Current Employer is not listed.

G. Kansiime reports the following:
Employer: Mbarara University of Science and Technology

I understand that the information above will be published within the journal article, if accepted, and that failure to comply and/or to accurately and completely report the potential financial conflicts of interest could lead to the following: 1) Prior to publication, article rejection, or 2) Post-publication, sanctions ranging from, but not limited to, issuing a correction, reporting the inaccurate information to the authors' institution, banning authors from submitting work to ASN journals for varying lengths of time, and/or retraction of the published work.

Name: Grace Kansiime
Manuscript ID: K360-2024-000147R1
Manuscript Title: Incidence of Acute Kidney Injury and Associated Mortality among Individuals with Drug-Susceptible Tuberculosis in Uganda
Date of Completion: May 24, 2024
Disclosure Updated Date: May 20, 2024

ASN American Society of Nephrology | JASN Journal of the American Society of Nephrology | KIDNEY360 Accessing Our World From Every Angle | CJASN Clinical Journal of the American Society of Nephrology

ASN Journal Disclosure Form

As per ASN journal policy, I have disclosed any financial relationships or commitments I have held in the past 36 months as included below. I have listed my Current Employer below to indicate there is a relationship requiring disclosure. If no relationship exists, my Current Employer is not listed.

M. Kanyesigye reports the following:
Employer: Mbarara University of Science and Technology

I understand that the information above will be published within the journal article, if accepted, and that failure to comply and/or to accurately and completely report the potential financial conflicts of interest could lead to the following: 1) Prior to publication, article rejection, or 2) Post-publication, sanctions ranging from, but not limited to, issuing a correction, reporting the inaccurate information to the authors' institution, banning authors from submitting work to ASN journals for varying lengths of time, and/or retraction of the published work.

Name: Michael Kanyesigye
Manuscript ID: K360-2024-000147R1
Manuscript Title: Incidence of Acute Kidney Injury and Associated Mortality among Individuals with Drug-Susceptible Tuberculosis in Uganda
Date of Completion: May 24, 2024
Disclosure Updated Date: May 24, 2024

ASN American Society of Nephrology | JASN Journal of the American Society of Nephrology | KIDNEY360 Accessing Our World From Every Angle | CJASN Clinical Journal of the American Society of Nephrology

ASN Journal Disclosure Form

As per ASN journal policy, I have disclosed any financial relationships or commitments I have held in the past 36 months as included below. I have listed my Current Employer below to indicate there is a relationship requiring disclosure. If no relationship exists, my Current Employer is not listed.

C. Muzoora has nothing to disclose.

I understand that the information above will be published within the journal article, if accepted, and that failure to comply and/or to accurately and completely report the potential financial conflicts of interest could lead to the following: 1) Prior to publication, article rejection, or 2) Post-publication, sanctions ranging from, but not limited to, issuing a correction, reporting the inaccurate information to the authors' institution, banning authors from submitting work to ASN journals for varying lengths of time, and/or retraction of the published work.

Name: Conrad Muzoora
Manuscript ID: K360-2024-000147R2
Manuscript Title: Incidence of Acute Kidney Injury and Associated Mortality among Individuals with Drug-Susceptible Tuberculosis in Uganda
Date of Completion: July 28, 2024
Disclosure Updated Date: July 28, 2024

ASN American Society of Nephrology JASN Journal of the American Society of Nephrology KIDNEY360 Accessing Our World From Every Angle CJASN Clinical Journal of the American Society of Nephrology

ASN Journal Disclosure Form

As per ASN journal policy, I have disclosed any financial relationships or commitments I have held in the past 36 months as included below. I have listed my Current Employer below to indicate there is a relationship requiring disclosure. If no relationship exists, my Current Employer is not listed.

F. Wilson reports the following:
Employer: Yale School of Medicine; Consultancy: Hekaheart; Aura.Care; Ownership Interest: Owner of Efference, LLC; Research Funding: Amgen; Vifor; Whoop; Advisory or Leadership Role: Editorial Board - American Journal of Kidney Disease; Editorial Board - Clinical Journal of the American Society of Nephrology; and Other Interests or Relationships: Medical columnist- Medscape.

I understand that the information above will be published within the journal article, if accepted, and that failure to comply and/or to accurately and completely report the potential financial conflicts of interest could lead to the following: 1) Prior to publication, article rejection, or 2) Post-publication, sanctions ranging from, but not limited to, issuing a correction, reporting the inaccurate information to the authors' institution, banning authors from submitting work to ASN journals for varying lengths of time, and/or retraction of the published work.

Name: Francis Perry Wilson
Manuscript ID: K360-2024-000147R2
Manuscript Title: Incidence of Acute Kidney Injury and Associated Mortality among Individuals with Drug-Susceptible Tuberculosis in Uganda
Date of Completion: July 28, 2024
Disclosure Updated Date: May 2, 2024

ASN
American Society of Nephrology
JASN
Journal of the American Society of Nephrology
KIDNEY360
Accessing Our World From Every Angle
CJASN
Clinical Journal of the American Society of Nephrology

ASN Journal Disclosure Form

As per ASN journal policy, I have disclosed any financial relationships or commitments I have held in the past 36 months as included below. I have listed my Current Employer below to indicate there is a relationship requiring disclosure. If no relationship exists, my Current Employer is not listed.

F. Yasmin has nothing to disclose.

I understand that the information above will be published within the journal article, if accepted, and that failure to comply and/or to accurately and completely report the potential financial conflicts of interest could lead to the following: 1) Prior to publication, article rejection, or 2) Post-publication, sanctions ranging from, but not limited to, issuing a correction, reporting the inaccurate information to the authors' institution, banning authors from submitting work to ASN journals for varying lengths of time, and/or retraction of the published work.

Name: Farah Yasmin
Manuscript ID: K360-2024-000147R2
Manuscript Title: Incidence of Acute Kidney Injury and Associated Mortality among Individuals with Drug-Susceptible Tuberculosis in Uganda
Date of Completion: August 1, 2024
Disclosure Updated Date: May 10, 2024
