# Supplementary material for: Incidence of Acute Kidney Injury and Associated Mortality among Individuals with Drug-Susceptible Tuberculosis in Uganda
Source: Kidney360. 2024 Aug 14;5(10):1446–54. doi: 10.34067/KID.0000000000000551 (PMC11556924; doi:10.34067/KID.0000000000000551)
Supplement: SUPPLEMENTARY MATERIAL [file kidney360-5-1446-s002.pdf]

The table of contents for the Supplemental Material **Page**

Table of contents……………………………………………………………………………………………1

Supplemental Table 1: Characteristics of participants by AKI category…..………………………………..2

Supplemental Figure 1: Follow up Data Collection Form………………………………………………..…4

Supplemental Figure 2: Trajectory of participants' serum creatinine from Baseline to follow up day 7…….8

Supplemental Table S1 Characteristics of Patients with Acute Kidney Injury

	AKI = 0	AKI = 1	p
n	99	52	
Age (median [IQR])	36.80 [27.96, 50.59]	41.26 [30.59, 55.53]	0.133
Female, n (%)<br>Male, n (%)	30 (30.3)<br>69 (69.7)	17 (32.7)<br>35 (67.3)	0.907
Education above primary, n (%)	40 (40.4)	21 (40.4)	1
Distance to site (median (IQR)), km	19.0 [6.0, 36.5]	24.0 [6.0, 41.5]	0.908
Residence type (%)			0.231
Rural, *n (%)*	47 (47.5)	29 (55.8)	
Suburban, *n (%)*	10 (10.1)	8 (15.4)	
Urban, *n (%)*	42 (42.4)	15 (28.8)	
Admitted to hospital, n (%)	42 (42.4)	30 (57.7)	0.107
Hypertension, n (%)	1 (1.0)	3 (5.8)	0.231
Diabetes mellitus, n (%)	4 (4.0)	3 (5.8)	0.942
HIV positive, n (%)	50 (50.5)	26 (50.0)	1
CD4 (median [IQR])	100.50 [43.00, 247.50]	75.50 [40.75, 126.25]	0.413
CD4 < 200, n %)	36 (72.0)	17 (73.9)	1.0
On HAART, n (%)	23 (46.0)	11 (42.3)	0.949
BMI (median [IQR])	18.00 [16.55, 20.20]	18.30 [16.15, 20.20]	0.779
BMI categories, n (%)			0.755
<18.5 kg/m^2	53 (53.5)	27 (51.9)	
18.5-25 kg/m^2	40 (40.4)	23 (44.2)	
> 25 kg/m^2	6 (6.0)	2 (3.8)	
Cough, n (%)	86 (86.9)	48 (92.3)	0.463
Hemoptysis, n (%)	14 (16.3)	6 (12.5)	0.737
Dyspnea, n (%)	37 (37.4)	22 (42.3)	0.678
Weight loss, n (%)	90 (90.9)	47 (90.4)	1
Weight loss, n (%)			0.79
≤2kg, n (%)	16 (16.2)	9 (17.3)	
2-5kg, n (%)	26 (26.3)	16 (30.8)	
>5kg, n (%)	57 (57.6)	27 (51.9)	
Pulmonary TB, n (%)	92 (92.9)	48 (92.3)	1
Duration on TB meds, (median [IQR])	0.00 [0.00, 1.00]	0.00 [0.00, 1.00]	0.406
Time since TB diagnosis (median [IQR])	1.00 [0.00, 1.00]	1.00 [0.00, 2.25]	0.116
pulse (median [IQR])	101.00 [91.00, 117.00]	101.00 [85.25, 117.25]	0.377

SBP (median [IQR])	109.00 [98.00, 120.00]	104.00 [95.75, 120.00]	0.209
DBP (median [IQR])	71.00 [64.00, 79.00]	69.50 [63.00, 76.00]	0.305
SPO2 (median [IQR])	97.0 [95.0, 99.0]	97.5 [95.0, 98.0]	0.472
Hemoglobin (median [IQR])	11.90 [9.45, 14.10]	10.50 [9.12, 13.62]	0.156
WBC count x 10^3 (median [IQR])	6.2 [4.5, 9.2]	5.7 [4.0, 8.6]	0.387
Neutrophils % (median [IQR])	65.59 [49.16, 75.35]	58.66 [46.61, 73.03]	0.184
Lymphocytes % (median [IQR])	20.86 [14.47, 35.00]	26.80 [19.02, 39.72]	0.028
Eosinophils % (median [IQR])	1.26 [0.32, 2.51]	1.17 [0.30, 3.24]	0.923
Monocytes % (median [IQR])	9.03 [6.25, 12.03]	9.01 [5.16, 11.85]	0.575
Platelet count x 10^3 (median [IQR])	289 [195.5, 398.5]	232.5 [146.3, 331.0]	0.024
Abnormal urinalysis = n (%)	29 (29.3)	29 (55.8)	0.003
BUN day 0 (median [IQR]), mg/dL	17.6 [12.3, 21.5]	21.4 [16.1, 37.3]	<0.001
Creatinine day 0 (median [IQR]), mg/dL	0.83 [0.72, 0.98]	1.02 [0.71, 1.53]	0.002
eGFR day 0, (median [IQR]), ml/min/1.73m^2	105.19 [88.04, 122.39]	67.29 [52.47, 120.18]	0.001
Creatinine day 7 (median [IQR]), mg/dL	0.80 [0.66, 0.94]	1.30 [1.05, 1.74]	<0.001
AKI creatinine (median [IQR]), mg/dL	NA	1.51 [1.23, 1.93]	NA
AKI stage at diagnosis, n (%)			
1		29 (55.8)	
2		19 (36.5)	
3		4 (7.7)	
Study site, n (%)			0.007
Mbarara	80 (80.8)	32 (61.5)	
Kabale	7 (7.1)	13 (25.0)	
Masaka	12 (12.1)	7 (13.5)	
Hematuria, n (%)	8 (8.1)	11 (21.6)	0.036
Proteinuria, n (%)	11 (11.1)	18 (34.6)	0.001

AKI, Acute Kidney Injury, BMI, body mass index, BUN, Blood Urea Nitrogen, CD4, clusters of differentiation4, DBP, diastolic blood pressure, eGFR estimated glomerular filtration rate, HAART, highly active antiretroviral therapy, , HIV human immunodeficiency virus, HR, heart rate, IQR, interquartile range, sCr, serum creatinine, SPB, systolic blood pressure, SPO2, saturation of peripheral Oxygen, TB, tuberculosis

Follow up Form

Record ID	______________________
STUDY SITE	◯ Mbarara (MBR)<br>◯ Kabale (KBL)<br>◯ Masaka (MSK)
Follow up visit	◯ day 7<br>◯ month 2<br>◯ month 3<br>◯ month 6<br>◯ month 12<br>◯ month 18<br>◯ month 24<br>◯ unscheduled
reason for visit	______________________

Demographics

Enrollment Date	______________________<br>(DD/MM/YYYY)
staff following up participant	______________________<br>(Staff Initials)
Date of data collection	______________________<br>(DD/MM/YYYY)
Date of data entry	______________________<br>(DD/MM/YYYY)
participant Initials	______________________<br>(write first then last initial)
Study ID	______________________

persistent Symptoms

	Yes	No
Do you have cough	○	○
Do you produce sputum	○	○
Do you cough up blood	○	○
Do you have shortness of breath	○	○
Have you lost weight since the treatment started	○	○

how much weight have you lost since starting treatment	○ none<br>○ about 2kg<br>○ 2-5kg<br>○ >5kg
duration since TB diagnosis	____________________
have you completed TB treatment	○ Yes<br>○ No
Duration since initiation of TB treatment	____________________<br>(Days/month)
any treatment interruption since last review	○ Yes<br>○ No<br>(Days)
duration of treatment interruption	____________________<br>(Days)
reason for treatment interruption	☐ Vomiting<br>☐ IRIS<br>☐ forgot<br>☐ liver injury<br>☐ AKI<br>☐ alcohol<br>☐ other
REPEAT SPUTUM TEST	○ POSITIVE<br>○ NEGATIVE<br>○ NOT YET<br>○ NOT APPLICABLE
Have you been admitted since last review	○ Yes<br>○ No
reason for admission/diagnosis	____________________
have you been discharged since last review	○ Yes<br>○ No

date of discharge	______________
have you noticed a change in your urine amount since starting TB treatment	◯ Yes<br>◯ No
about how much urine do you pass in 24 hrs	◯ < 100ml<br>◯ 100-500ml<br>◯ >500ml

	Yes	No
do you have facial swelling	◯	◯
do you have lower limb swelling	◯	◯

medication history

TB treatment regimen	◯ RHZE<br>◯ RH<br>◯ Other<br>(Specify other)
have you initiated any new treatment since last review	◯ Yes<br>◯ No
type of new treatment started	◯ HAART<br>◯ DM meds<br>◯ HTN meds<br>◯ antibiotics<br>◯ herbs<br>◯ others

nephrotoxic meds

	Yes	No
Patient taking any other meds that cause nephrotoxicity	◯	◯

Physical examination

pallor	◯ none<br>◯ mild<br>◯ moderate<br>◯ severe
oedema	◯ none<br>◯ Ankle<br>◯ Below knee<br>◯ Anasarca
weight	______________<br>(kgs)
height	______________<br>(cm)

BMI	________________
pulse rate	________________ (bpm)
systolic Blood pressure	________________ (mmHg)
diastolic blood pressure	________________ (mmHg)
respiratory rate	________________ (breaths per minute)
oxygen saturation	________________ (percent)
TB TREATEMENT OUTCOME	◯ CURED<br>◯ COMPLETED<br>◯ DIED<br>◯ LOST TO FOLLOW UP<br>◯ FAILED<br>◯ NOT EVALUATED
DATE OF DEATH	________________

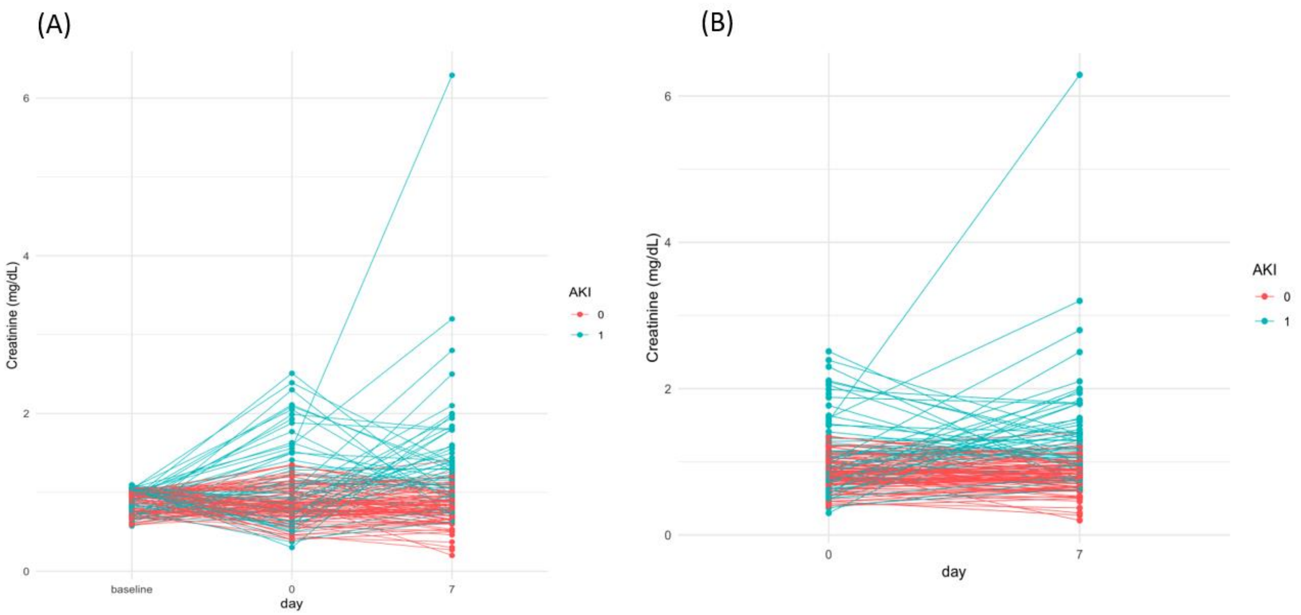

Figure supplement S2: A, Trajectory of creatinine from an imputed Baseline to day 0 and day 7; B, Trajectory of creatinine from day 0 to follow up day 7

AKI; acute kidney injury.
